# Supplementary material for: LncRNA PNKY Is Upregulated in Breast Cancer and Promotes Cell Proliferation and EMT in Breast Cancer Cells
Source: Noncoding RNA. 2023 Apr 6;9(2):25. doi: 10.3390/ncrna9020025 (PMC10143469; doi:10.3390/ncrna9020025)
Supplement: Supplementary file 1 [file ncrna-09-00025-s001.zip › ncrna-2029857-supplementary.pdf]

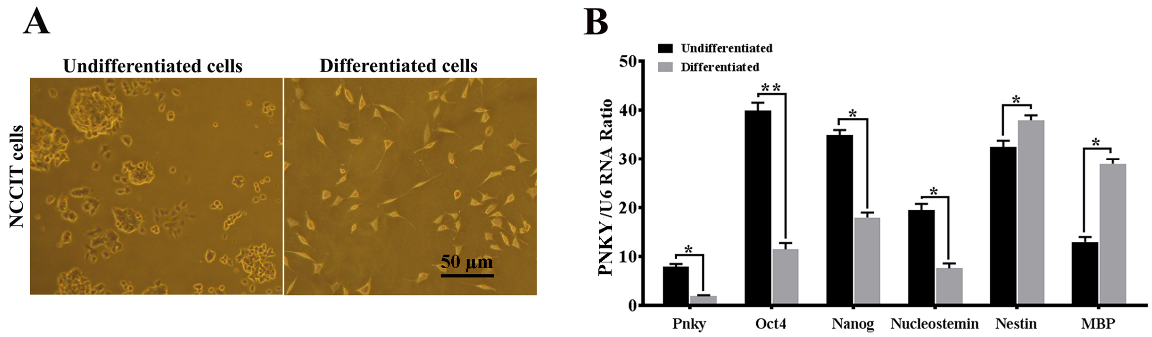

Supplementary Figure S1: PNKY expression is downregulated during neural differentiation. (**A**) undifferentiated (left) and differentiated (right) NCCIT cells. (**B**) The expression level of PNKY, like the expression level of stemness factors including Oct4/ Nanog /Nucleostemin, is downregulated during differentiation of NCCIT cells. The values shown represent the mean ± SE. * *p* value < 0.05, ** *p* value < 0.01.
